# Pain control following total hip arthroplasty: a prospective randomized controlled trial comparing spinal anaesthesia without adjuncts versus fascia iliaca block versus pericapsular nerve group block versus local anaesthetic infiltration

**DOI:** 10.1302/2633-1462.75.BJO-2026-0003.R1

**Published:** 2026-05-07

**Authors:** J. Patrick Park, Kevin Yan Zhao, Tanya Cierson, Bardia Barimani, Eric Belzile, De Q. Tran, Anthony Albers

**Affiliations:** 1 Division of Orthopaedic Surgery, McGill University, Montreal, Canada; 2 St-Mary’s Hospital Center and Division of Orthopaedic Surgery, Montreal, Canada; 3 Faculty of Medicine and Health Sciences, McGill University, Montreal, Canada; 4 Department of Orthopaedic Surgery and Rehabilitation, University of Texas Medical Branch, Galveston, Texas, USA; 5 St-Mary’s Hospital Research Centre, Montreal, Canada; 6 Department of Anesthesiology, McGill University, Montreal, Canada

**Keywords:** Total hip arthroplasty, Multimodal analgesia, Pain control, Nerve block, Opioid consumption, Patient satisfaction, Total hip arthroplasty (THA), spinal anaesthesia, local anaesthetic, fascia iliaca, nerve, randomized controlled trial, visual analogue scale (VAS), opioids, postoperative pain, primary total hip arthroplasty

## Abstract

**Aims:**

Postoperative pain management for total hip arthroplasty (THA) remains an area for improvement. This study compared the impact of suprainguinal fascia iliaca block (FIB) versus local anaesthetic infiltration (LAI), versus pericapsular nerve group (PENG) block, and versus spinal anaesthesia alone, on early postoperative pain in patients who underwent inpatient primary THA.

**Methods:**

This was a single-centre, assessor- and participant-blinded randomized controlled trial. A total of 240 patients undergoing THA under spinal anaesthesia were randomized to LAI, FIB, PENG block, or control in a 1:1:1:1 ratio. The primary outcome was pain at four hours postoperatively (visual analogue scale (VAS)). Secondary outcomes included VAS at other timepoints, opioid consumption, patient satisfaction, and length of hospital stay (LOS). A VAS difference of 2 cm was considered clinically significant.

**Results:**

A total of 240 participants were randomized, with 222 in the final analysis. Only LAI (mean VAS 1.6 (SD 2.2)) significantly decreased postoperative pain compared with the control group (mean VAS 3.0 (SD 2.7); p = 0.004) at four hours. VAS at four hours was not significantly different between LAI, FIB, and PENG block groups in direct comparisons. FIB (23.3 mg morphine equivalents (MEQ) (SD 18.4), p = 0.001) and LAI (22.2 mg MEQ (SD 18.7), p = 0.001) significantly decreased opioid consumption compared with the control group (36.7 mg MEQ (SD 24.5)) at 24 hours postoperatively. The same was found at 48 hours postoperatively. LAI significantly improved patient satisfaction scores at four hours compared with the control group (1.3 (SD 0.6) vs 1.9 (SD 1.2), p = 0.043). There was no difference in LOS between study groups. One patient had femoral nerve motor deficit following PENG block, with full recovery at six months postoperatively.

**Conclusion:**

LAI combined with spinal anaesthesia reduced early postoperative pain and improved patient satisfaction with pain control compared with spinal anaesthesia alone. LAI and FIB both decreased opioid consumption in the first 24 and 48 hours.

Cite this article: *Bone Jt Open* 2026;7(5):601–612.

## Introduction

Total hip arthroplasty (THA) is a commonly performed procedure for the treatment of advanced hip osteoarthritis.^1^ Early postoperative pain control remains an area for improvement in THA, being one of the main causes of failure of same-day hospital discharge.^2^

Multimodal analgesia is the current gold standard to optimize perioperative pain, as outlined in the PROcedure SPECific Pain management (PROSPECT) guidelines.^3-5^ It includes acetaminophen; cyclo-oxygenase-2-selective inhibitors or non-steroidal anti-inflammatory drugs; intraoperative dexamethasone; analgesic adjuncts such as suprainguinal fascia iliaca block (FIB) or local anaesthetic infiltration (LAI); and opioids. FIB involves anaesthetic injection under the fascia of the iliacus muscle to provide sensory blockade to the femoral, lateral femoral cutaneous, and obturator nerves.^6^ Additionally, recent studies have reported that pericapsular nerve group (PENG) block reduces opioid consumption perioperatively.^7-9^ PENG block involves injecting local anaesthetic in the myofascial plane between the psoas muscle and the superior pubic ramus to target the sensory nerve branches to the anterior joint capsule, which include branches of the femoral, obturator, and accessory obturator nerves.^10^

To date, comparison of these methods of analgesic adjuncts has been limited, with heterogeneous results.^11-17^ Existing studies in this area are limited by various factors, including direct comparison of pain control adjuncts without a control group. Further, there exist variations in surgical approach, the use of general or spinal anaesthesia, timing of pain control adjunct administration (preoperatively, intraoperatively, or postoperatively), and medications or dosages used in pain control adjuncts that limit direct comparison of multiple pain control adjuncts across studies.^11-17^ As such, a comparative assessment of these analgesic methods is of vital importance to guide future practice. To our knowledge, this is the first prospective randomized controlled trial directly comparing the impact of FIB versus LAI versus PENG block versus spinal anaesthesia alone without the use of an adjunct on early postoperative pain in patients undergoing elective, inpatient primary THA.

## Methods

### Ethics and funding

Ethics approval was obtained from the Institutional Research Ethics Board (REB) of the St-Mary’s Hospital Research Centre. Informed consent was obtained from all participants prior to surgery. This project was funded by the St-Mary’s Hospital Foundation, through the CARE grant research programme of the St-Mary’s Hospital Research Centre. The clinical trial registration number is NCT05062356.

### Inclusion and exclusion criteria

The enrolment lasted from November 2021 to July 2024. Inclusion and exclusion criteria are outlined in Table I. If a patient withdrew consent, all collected data were discarded and excluded from analyses.

**Table I.** Inclusion and exclusion criteria.

Inclusion criteria	Exclusion criteria
Patients aged ≥ 18 years who required elective, inpatient primary total hip arthroplasty for the treatment of advanced osteoarthritis	Previous fracture or surgery to the affected hip Anaesthesia use other than spinal BMI > 45 kg/m^2^ Allergies to the medications administered as part of the study protocol Preoperative daily consumption of opioid analgesics Unable to provide informed consent for himself/herself due to a pre-existing cognitive impairment

### Study design and procedures

This study is a prospective randomized control trial, with participants randomized to one of three intervention groups (LAI, FIB, and PENG block), or the control group (spinal anaesthesia alone without adjunct). Participants received an information booklet guiding them through relevant details of the surgery. A computer-generated blocked randomization sequence, with a block size of six, was used to randomize participants in a 1:1:1:1 fashion. The randomization result was communicated to the surgeon and anaesthesiologist via sealed envelope. Postoperatively, the nursing team and physiotherapists were blinded to the participant’s study arm.

All THA procedures were conducted by fellowship-trained orthopaedic surgeons (AA; other participating surgeons included in the Acknowledgements) using a posterior approach. Every patient received spinal anaesthesia using 2.5 ml of bupivacaine 0.5% and appropriate sedation without intravenous pain control. When LAI was given, it consisted of 50 ml bupivacaine hydrochloride 0.2% with epinephrine 1:200,000 and 15 mg ketorolac. The surgeon performed the injection in the anterior pericapsular tissues, avoiding areas near the sciatic nerve, as well as the fascia lata and subcutaneous tissues. If FIB or PENG block was performed following skin closure, a surgical drape was maintained to obstruct the patient’s view. FIB included 50 ml bupivacaine hydrochloride 0.2% with epinephrine 1:200,000, and PENG block included 20 ml bupivacaine hydrochloride 0.5% with epinephrine 1:200,000. Patients not receiving LAI received 15 mg of intramuscular ketorolac to account for the effect of systemic absorption of locally injected ketorolac on pain control. All FIB and PENG blocks were performed by staff anaesthesiologists or supervised anaesthesiology trainees with ultrasound guidance, using reproducible sonological endpoints: separation of the plane between the fascia iliaca and the iliacus muscle for the FIB and lifting of the psoas tendon for the PENG block.

### Patient demographic characteristics

**Fig. 1:**
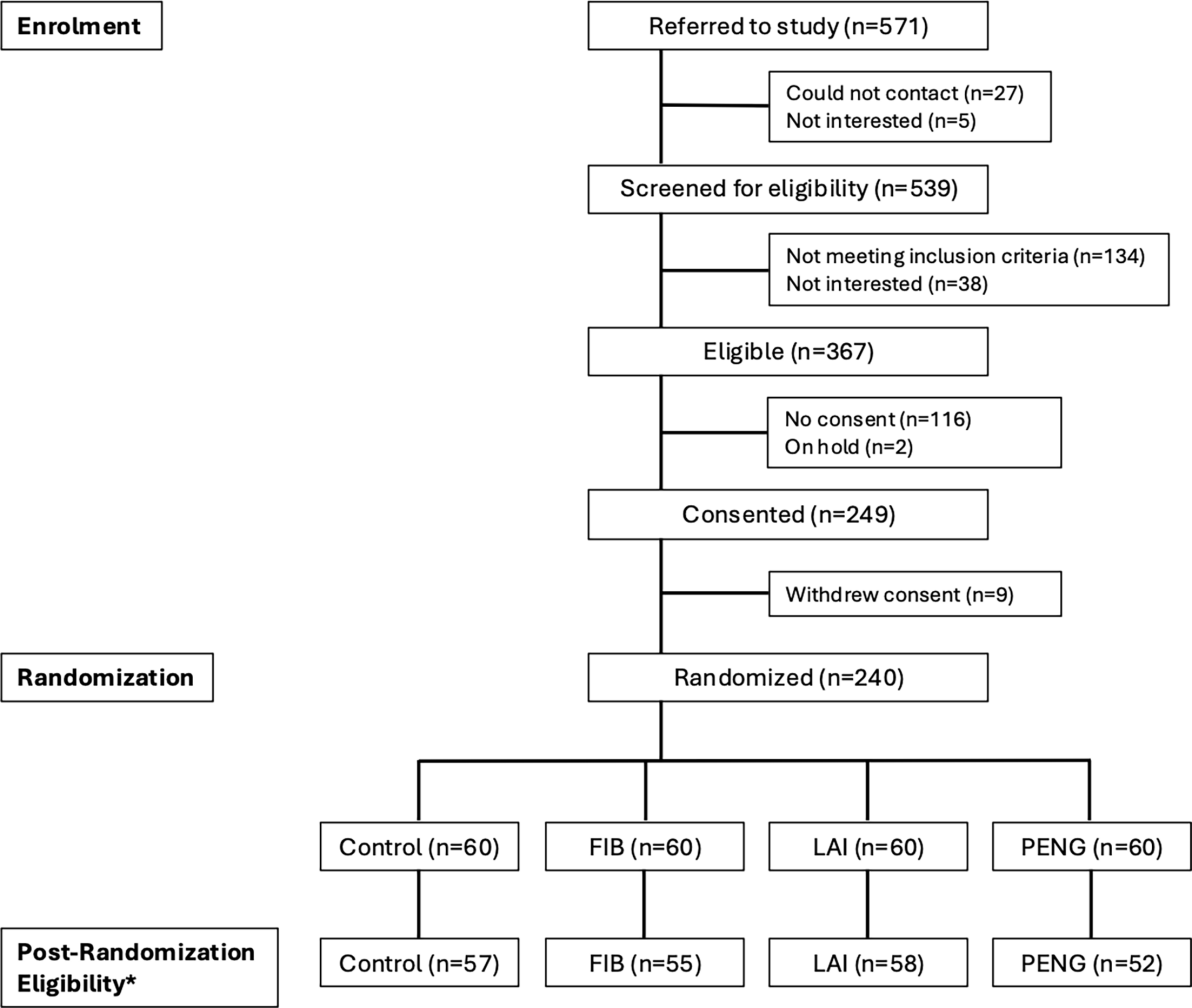
Flowchart of enrolment and randomization for the trial. *Reasons for non-eligibility/exclusion: incorrect adjunct or no adjunct given (n = 6), required general anaesthesia at the time of surgery (n = 5), surgical approach other than posterior approach (n = 3), BMI > 45 kg/m^2^ (n = 1), traumatic injury post-surgery (n = 1), other (n = 2). FIB, fascia iliaca block; LAI, local anaesthetic infiltration; PENG, pericapsular nerve group.

A total of 240 participants were recruited, with 222 participants in the final analysis after exclusions (Figure 1). Patient demographic characteristics were evenly distributed across the study arms, with minor differences that were not clinically relevant (Table II). The mean participant age was 71.3 years (SD 8.8), with a comparable ratio of sex across study groups.

**Table II.** Baseline variables by study group.

Baseline variables	Overall	Control	FIB	LAI	PENG	Average standardized difference†
	(n = 222)	(n = 57)	(n = 55)	(n = 58)	(n = 52)	
**Demographic**						
Mean age, yrs (SD)	71.3 (8.8)	69.6 (9.9)	73.0 (8.1)	71.2 (8.1)	71.6 (8.8)	0.20
Female, n (%)	111 (50.0)	25 (43.9)	25 (45.5)	32 (55.2)	29 (55.8)	0.15
**Medical**						
Mean BMI, kg/m^2^ (SD)	28.4 (5.3)	29.4 (6.3)	28.6 (4.7)	28.0 (4.5)	27.6 (5.4)	0.19
Smoker, n (%)	16 (7.2)	4 (7.0)	6 (10.9)	3 (5.2)	3 (5.9)	0.12
Diabetes, n (%)	43 (19.5)	11 (19.3)	12 (21.8)	13 (22.4)	7 (13.7)	0.12
Anticoagulated (preop), n (%)	58 (27.0)	19 (34.6)	12 (23.1)	11 (19.6)	16 (30.8)	**0.20**
Alcohol consumption, one drink per day or more, n (%)*	26 (11.7)	9 (15.8)	6 (10.9)	4 (6.9)	7 (13.5)	0.16
Recreational drug use, n (%)	11 (5.0)	1 (1.8)	1 (1.8)	2 (3.5)	7 (13.5)	**0.25**
**Surgical**						
**Side of surgery, n (%)**						0.13
Left	104 (47.1)	25 (43.9)	30 (54.6)	25 (43.1)	24 (47.1)	
Right	117 (52.9)	32 (56.1)	25 (45.4)	33 (56.9)	27 (52.9)	
Previous joint arthroplasty, n (%)	73 (33.0)	20 (35.1)	20 (36.4)	16 (27.6)	17 (33.3)	0.10
Mean LOS, days (SD)	3.0 (2.7)	3.3 (3.3)	3.1 (2.7)	2.8 (2.3)	2.9 (2.3)	0.11
Mean duration of surgery, mins (SD)	96.0 (20.9)	97.7 (20.0)	97.0 (23.2)	95.8 (21.3)	93.1 (18.9)	0.12

Significant average standardized differences (> 0.2) are indicated in bold.

* Responses indicating ‘occasional’, ‘rarely’, or ‘very rarely’ were coded as < one drink per day.

† Standardized differences computed for each paired comparison and average of all paired comparisons.

FIB, fascia iliaca block; LAI, local anaesthetic infiltration; LOS, length of stay; OR, operating room; PENG, pericapsular nerve group block.

### Outcomes and data collection

The primary outcome was pain measured using the visual analogue scale (VAS)^18^ at four hours postoperatively. A VAS difference of 2 cm with a SD of 3 cm was considered clinically significant. The secondary outcomes were: 1) VAS immediately before and after the first physiotherapy session, on postoperative day (POD) 2, and on POD 7; 2) postoperative opioid consumption at four, 24, and 48 hours, and between 24 and 48 hours; and 3) postoperative patient satisfaction with pain control on a five-point Likert scale at four hours, immediately before and after the first physiotherapy session, on POD 2, and on POD 7, length of hospital stay (LOS), and complications (urinary retention, nausea/vomiting, pruritus/allergy, infection, or other).

The following demographic data were collected: age, sex, BMI, smoking status, alcohol consumption, recreational drug use, anticoagulant use, preoperative medications, diagnosis of diabetes, side of surgery, and previous hip or knee arthroplasty. The patient’s motor and sensory exam findings were documented by a resident physician on POD 1 to 3 (Supplementary Material, Appendix 1). The in-hospital questionnaire was completed by the patient at four hours postoperatively, immediately before and after the first physiotherapy session, on POD 2, and on POD 7 (Supplementary Material, Appendix 2). The nursing team recorded the participant’s neurological exam at four hours postoperatively. Upon discharge, participants independently completed the at-home questionnaire, which collected information on pain level, satisfaction with pain control, and pain medication use (Supplementary Material, Appendix 3).

### Statistical analysis

The sample size was calculated based on the primary outcome (VAS at four hours). A sample size of 240 patients was required based on the following parameters: one-way analysis of variance (ANOVA) test with a VAS difference of 2 cm (SD 3), *α* = 0.05, and power of 80% (calculated Cohen’s *f* effect size = 0.22). The Cohen’s *f* effect size of 0.22 represents a moderate effect size; for paired comparisons of study arms, a moderate effect size defined as Cohen’s *d* = 0.5 represents a mean difference of 1.5 cm under the same assumptions used for the one-way ANOVA.^19^

To assess baseline balance across study arms, standardized differences were calculated for each paired comparison.^20^ In total, 11 baseline covariates (age, sex, BMI, smoking, diabetes, preoperative anticoagulation, alcohol/drug consumption, hip location, previous joint arthroplasty, LOS, and operating time) were assessed. A standardized difference of ≥ 0.2 was defined as baseline imbalance.

For continuous outcomes, a one-way ANOVA (F-test) was used to test the equality of means of the four study arms. This four-arm trial was designed for direct comparison of unrelated interventions with a control (CTL) arm (FIB-CTL, LAI-CTL, PENG-CTL). As outlined by a published extension of the Consolidated Standards of Reporting Trials (CONSORT) 2010 statement,^21^ the interpretation of the results from one comparison generally does not have bearing on the interpretation of others in this case; therefore, no correction for multiple testing was performed.

Additional comparisons were performed to explore differences between pairs of intervention groups (FIB-LAI, FIB-PENG, LAI-PENG); and one overall comparison where all intervention groups were pooled together compared with the control group. For comparisons between pairs of intervention groups, independent-samples *t*-test was performed and the effect size was calculated as the mean difference divided by the pooled SD.^19^ The effect size adjusted for covariates (baseline imbalance) was calculated from the ‘β’ estimate of the intervention group in the linear regression model divided by the pooled SD obtained from the unadjusted analysis.^22,23^

Multiple imputation was conducted for the primary outcome to ensure the validity of the results.^24,25^ The linear regression adjusting for baseline imbalance and multiple imputation approach was only performed for the primary outcome (VAS pain at four hours) (Supplementary Table i). All analyses were performed using Stata v. 18.0 (StataCorp, USA). The threshold for statistical significance was p < 0.05.

## Results

### Postoperative pain

**Fig. 2:**
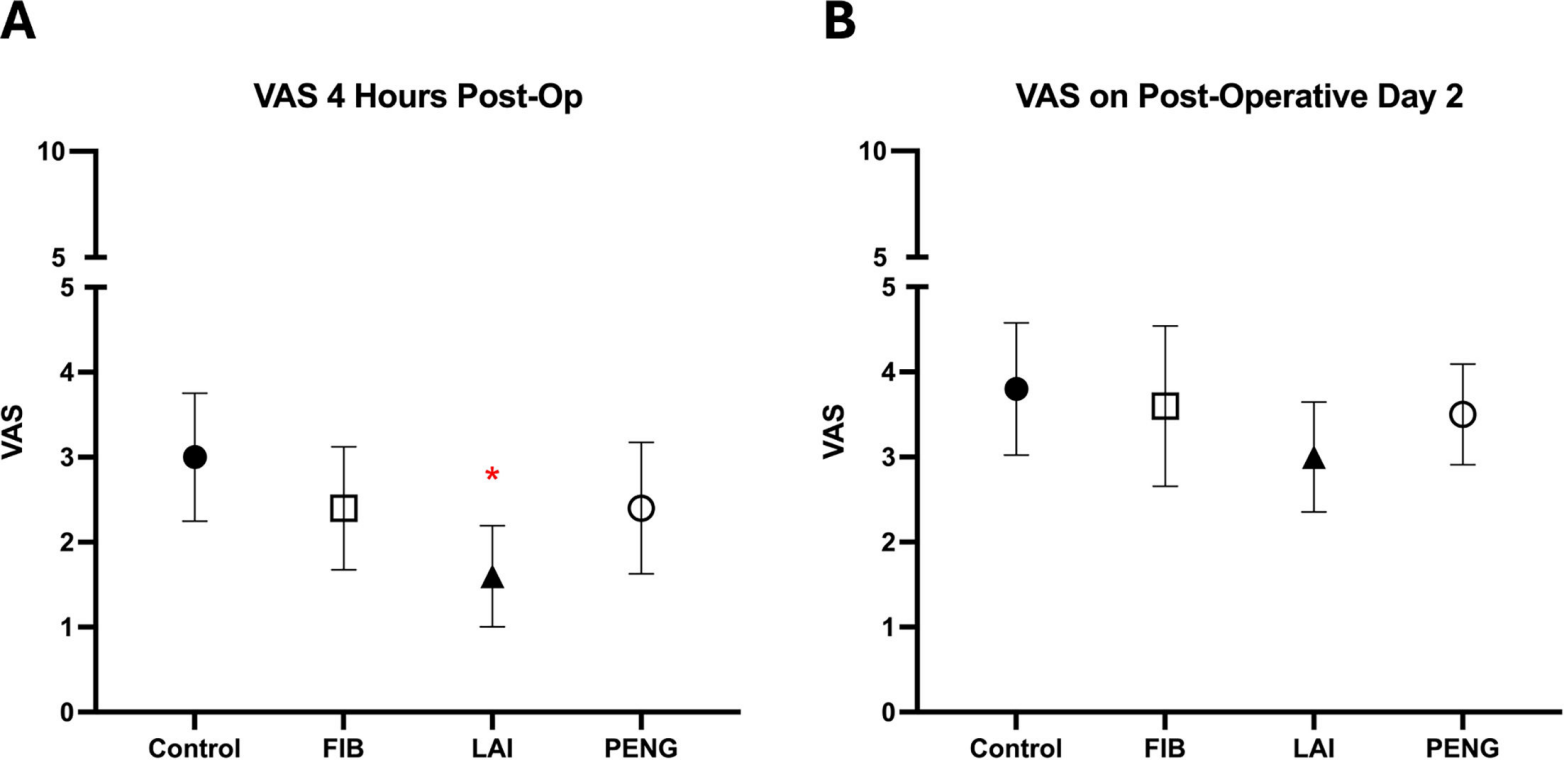
Level of pain measured with visual analogue scale (VAS) at a) four hours following surgery and b) two days following surgery. *Statistically significant (LAI significantly reduced pain compared with the control group, p =0.004, independent samples *t*-test). FIB, fascia iliaca block; LAI, local anaesthetic infiltration; PENG, pericapsular nerve group block.

At four hours postoperatively (Figure 2a, Table III and Table IV), LAI significantly reduced pain (VAS 1.6 (SD 2.2)) compared with the control group (VAS 3.0 (SD 2.7), p = 0.004, independent samples *t*-test), while FIB (2.4 (SD 2.6), p = 0.213, independent samples *t*-test) and PENG block (2.4 (SD 2.6), p = 0.280, independent samples *t*-test) showed no improvement.

**Table III.** Primary and secondary outcomes.

Outcome	Control (n = 57)	FIB (n = 55)	LAI (n = 58)	PENG (n = 52)	p-value*
**Primary outcome**					
Mean VAS, 4 hrs postop (SD)	3.0 (2.7)	2.4 (2.6)	1.6 (2.2)	2.4 (2.6)	0.047
**Secondary outcomes**					
**Mean VAS (SD)**					
Pre-physio	3.4 (2.5)	2.5 (2.8)	2.5 (2.3)	2.6 (1.9)	0.388
Post-physio	4.3 (2.8)	3.1 (2.2)	3.1 (1.8)	3.9 (2.4)	0.208
POD 2	3.8 (2.4)	3.6 (2.7)	3.0 (2.1)	3.5 (1.9)	0.473
POD 7	2.4 (1.9)	2.3 (2.2)	2.3 (2.0)	2.9 (1.7)	0.567
**Mean MEQ, mg (SD)**					
4 hrs postop	5.2 (8.1)	2.7 (6.4)	3.1 (5.8)	3.0 (6.9)	0.174
0 to 24 hrs	36.7 (24.5)	23.3 (18.4)	22.2 (18.7)	29.7 (17.1)	< 0.001
0 to 48 hrs	60.0 (42.2)	36.5 (27.3)	39.0 (30.1)	49.3 (29.1)	0.002
24 to 48 hrs	22.8 (23.1)	13.6 (14.0)	16.2 (16.8)	19.0 (15.6)	0.071
**Mean satisfaction (SD)**					
4 hrs postop	1.9 (1.2)	1.7 (0.9)	1.3 (0.6)	2.0 (1.4)	0.108
Pre-physio	2.1 (1.3)	1.8 (1.1)	1.7 (1.1)	2.0 (1.2)	0.764
Post-physio	2.4 (1.6)	2.2 (1.3)	1.9 (1.3)	2.4 (1.6)	0.779
POD 2	2.0 (1.3)	1.5 (0.8)	1.5 (0.9)	1.6 (0.9)	0.054
POD 7	1.6 (1.1)	1.5 (1.0)	1.5 (1.0)	1.6 (0.8)	0.955

* One-way analysis of variance.

FIB, fascia iliaca block; LAI, local anaesthetic infiltration; MEQ, morphine equivalent; PENG, pericapsular nerve group block; POD, postoperative day; VAS, visual analogue scale.

**Table IV.** Effect size and 95% CI for all paired comparisons for primary and secondary outcomes.

Outcomes	Main analyses			Additional analyses			
	Comparison with the control group			Overall intervention	Comparison between intervention groups		
	FIB/CTL	LAI/CTL	PENG/CTL	INT/CTL	FIB/LAI	FIB/PENG	LAI/PENG
**Primary outcomes**							
VAS, 4 hrs postop	0.25 (-0.14 to 0.63) p = 0.213	0.56 (0.18 to 0.95) p = 0.004*	0.22 (-0.18 to 0.62) p = 0.280	0.35 (0.04 to 0.67) p = 0.029*	-0.30 (-0.68 to 0.08) p = 0.121	0.03 (-0.37 to 0.42) p = 0.894	0.34 (-0.06 to 0.73) p = 0.097
**Secondary outcomes**							
**VAS**							
Pre-physio	0.37 (-0.17 to 0.90) p = 0.182	0.40 (-0.16 to 0.97) p = 0.163	0.38 (-0.17 to 0.94) p = 0.179	0.39 (-0.05 to 0.83) p = 0.082	0.00 (-0.57 to 0.57) p = 0.993	0.04 (-0.52 to 0.60) p = 0.881	0.05 (-0.53 to 0.63) p = 0.862
Post-physio	0.47 (-0.09 to 1.02) p = 0.103	0.50 (-0.08 to 1.07) p = 0.078	0.15 (-0.44 to 0.73) p = 0.625	0.41 (-0.05 to 0.86) p = 0.080	0.00 (-0.57 to 0.59) p = 0.979	0.34 (-0.27 to 0.94) p = 0.274	0.38 (-0.24 to 0.99) p = 0.236
POD 2	0.11 (-0.35 to 0.57) p = 0.647	0.35 (-0.09 to 0.79) p = 0.118	0.16 (-0.28 to 0.60) p = 0.469	0.21 (-0.15 to 0.58) p = 0.250	-0.21 (-0.66 to 0.24) p = 0.360	-0.03 (-0.48 to 0.42) p = 0.899	0.21 (-0.21 to 0.64) p = 0.319
POD 7	0.04 (-0.48 to 0.57) p = 0.876	0.09 (-0.41 to 0.58) p = 0.732	-0.25 (-0.74 to 0.24) p = 0.319	-0.04 (-0.45 to 0.37) p = 0.841	-0.04 (-0.55 to 0.47) p = 0.883	0.27 (-0.24 to 0.78) p = 0.293	0.33 (-0.14 to 0.81) p = 0.172
**MEQ**							
4 hrs postop	0.35 (-0.02 to 0.72) p = 0.066	0.31 (-0.06 to 0.67) p = 0.103	0.30 (-0.08 to 0.68) p = 0.118	0.34 (0.04 to 0.64) p = 0.041*	0.07 (-0.30 to 0.43) p = 0.728	0.04 (-0.34 to 0.42) p = 0.820	-0.02 (-0.39 to 0.36) p = 0.929
0 to 24 hrs	0.62 (0.24 to 0.99) p = 0.001*	0.66 (0.29 to 1.04) p = 0.001*	0.33 (-0.05 to 0.71) p = 0.085	0.59 (0.28 to 0.89) p = 0.001*	-0.06 (-0.43 to 0.31) p = 0.763	0.36 (-0.03 to 0.74) p = 0.068	0.41 (0.03 to 0.79) p = 0.033
0 to 48 hrs	0.66 (0.25 to 1.07) p = 0.002*	0.57 (0.15 to 0.98) p = 0.007*	0.29 (-0.12 to 0.70) p = 0.163	0.57 (0.23 to 0.90) p = 0.007*	0.09 (-0.31 to 0.48) p = 0.666	0.46 (0.04 to 0.87) p = 0.030	0.35 (-0.07 to 0.76) p = 0.102
24 to 48 hrs	0.48 (0.08 to 0.89) p = 0.021*	0.33 (-0.08 to 0.73) p = 0.119	0.19 (-0.22 to 0.60) p = 0.363	0.37 (0.04 to 0.71) p = 0.041*	0.16 (-0.23 to 0.57) p = 0.412	0.36 (-0.04 to 0.77) p = 0.079	0.17 (-0.24 to 0.59) p = 0.410
**Satisfaction**							
4 hrs postop	0.15 (-0.39 to 0.70) p = 0.585	0.61 (0.04 to 1.18) p = 0.043*	-0.07 (-0.61 to 0.47) p = 0.810	0.19 (-0.26 to 0.65) p = 0.409	-0.54 (-1.08 to 0.01) p = 0.052	0.22 (-0.31 to 0.74) p = 0.414	0.63 (0.08 to 1.17) p = 0.020*
Pre-physio	0.22 (-0.35 to 0.79) p = 0.448	0.26 (-0.34 to 0.87) p = 0.397	0.05 (-0.52 to 0.62) p = 0.867	0.18 (-0.29 to 0.65) p = 0.451	-0.05 (-0.65 to 0.55) p = 0.872	0.18 (-0.39 to 0.75) p = 0.539	0.23 (-0.38 to 0.84) p = 0.468
Post-physio	0.12 (-0.44 to 0.67) p = 0.674	0.29 (-0.31 to 0.90) p = 0.346	0.02 (-0.56 to 0.60) p = 0.942	0.14 (-0.32 to 0.60) p = 0.547	-0.20 (-0.81 to 0.42) p = 0.529	0.10 (-0.50 to 0.69) p = 0.749	0.27 (-0.37 to 0.91) p = 0.406
POD 2	0.51 (0.03 to 0.99) p = 0.034*	0.48 (0.03 to 0.93) p = 0.042*	0.43 (-0.02 to 0.88) p = 0.072	0.53 (0.15 to 0.91) p = 0.030*	0.02 (-0.44 to 0.48) p = 0.935	0.11 (-0.35 to 0.57) p = 0.648	0.08 (-0.35 to 0.51) p = 0.713
POD 7	0.11 (-0.44 to 0.65) p = 0.705	0.04 (-0.47 to 0.55) p = 0.884	-0.03 (-0.53 to 0.47) p = 0.904	0.03 (-0.39 to 0.46) p = 0.875	0.07 (-0.46 to 0.60) p = 0.792	0.16 (-0.36 to 0.68) p = 0.547	0.08 (-0.40 to 0.56) p = 0.755

Positive value indicates in favour of the 1st group label. Negative value indicates in favour of the 2nd group label.

* Statistically significant effect size.

FIB, fascia iliaca block; INT, interventions group (FIB, LAI, PENG pooled together); LAI, local anaesthetic infiltration; MEQ, morphine equivalent; PENG, pericapsular nerve group block; POD, postoperative day; VAS, visual analogue scale.

Before and after the first physiotherapy session, and on POD 2 and POD 7, no analgesic adjunct resulted in improved VAS (p > 0.05). Similarly, there were no significant differences in VAS between pairs of intervention groups (p > 0.05).

For the primary outcome (VAS at four hours), similar results were observed when comparing the different study groups with each other rather as with the control group (FIB/LAI, p = 0.121; FIB/PENG, p = 0.894; LAI/PENG, p = 0.097, independent samples *t*-test) (Table IV). The effect size adjusted for baseline imbalance was presented and a multiple imputation approach was performed (Supplementary Table i).

### Opioid consumption

Cumulative opioid consumption was measured in morphine equivalents (MEQ) over four, 24, and 48 hours (Table III and Table IV).

At four hours, FIB (2.7 (SD 6.4), p = 0.066), LAI (3.1 (SD 5.8), p = 0.103), and PENG block (3.0 (SD 6.9), p = 0.118) did not reduce opioid consumption compared with the control group (5.2 (SD 8.1), independent samples *t*-test). There were no significant differences between intervention groups (FIB/LAI, p = 0.728; FIB/PENG, p = 0.820; LAI/PENG, p = 0.929).

**Fig. 3:**
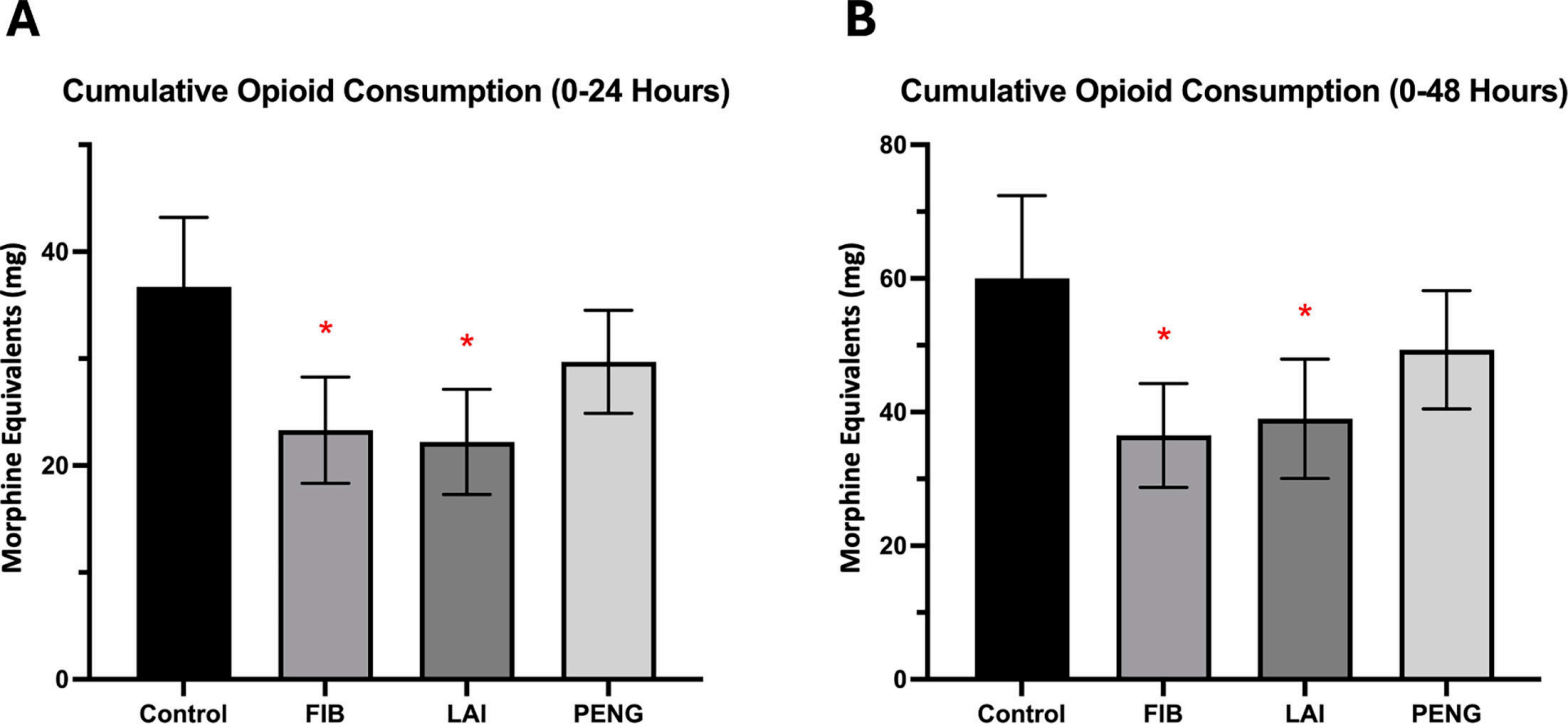
Cumulative postoperative opioid consumption in morphine equivalents at a) 24 hours following surgery and b) 48 hours following surgery. *Statistically significant. FIB, fascia iliaca block; LAI, local anaesthetic infiltration; PENG, pericapsular nerve group block.

At 24 hours, FIB (23.3 (SD 18.4), p = 0.001) and LAI (22.2 (SD 18.7), p < 0.001) reduced opioid consumption compared with the control group (36.7 (SD 24.5)), while PENG block showed no reduction (29.7 (SD 17.1), p = 0.084) (Figure 3a). LAI significantly reduced opioid consumption at 24 hours compared with PENG (p = 0.033). There was no significant difference between other pairs of interventions (FIB/LAI, p = 0.763; FIB/PENG, p = 0.068).

At 48 hours, participants who received FIB (36.5 (SD 27.3), p = 0.002) or LAI (39.0 (SD 30.1), p = 0.007) had decreased cumulative opioid consumption compared with the control group (60.0 (SD 42.2)). Participants who received PENG block had similar cumulative opioid consumption (49.3 (SD 29.1), p = 0.163) to the control group. FIB significantly reduced opioid consumption at 48 hours compared with the PENG block group (p = 0.030). There was no significant difference between other pairs of interventions (FIB/LAI, p = 0.666; LAI/PENG, p = 0.102).

Between 24 and 48 hours postoperative, FIB (13.6 (SD 14.0), p = 0.021) significantly reduced opioid consumption compared with the control group (22.8 (SD 23.1)). There was no significant difference between LAI (16.2 (SD 16.8), p = 0.119) or PENG block (19.0 (SD 15.6), p = 0.363) and the control group. There were no significant differences between intervention groups (FIB/LAI, p = 0.412; FIB/PENG, p = 0.079; LAI/PENG, p = 0.410).

### Patient satisfaction with pain control

Satisfaction with pain control at four hours postoperatively, before and after the first physiotherapy session, and on POD 2 and POD 7 was measured as: 1 = very satisfied; 2 = somewhat satisfied; 3 = neither satisfied nor dissatisfied; 4 = somewhat dissatisfied; 5 = very dissatisfied.

**Fig. 4:**
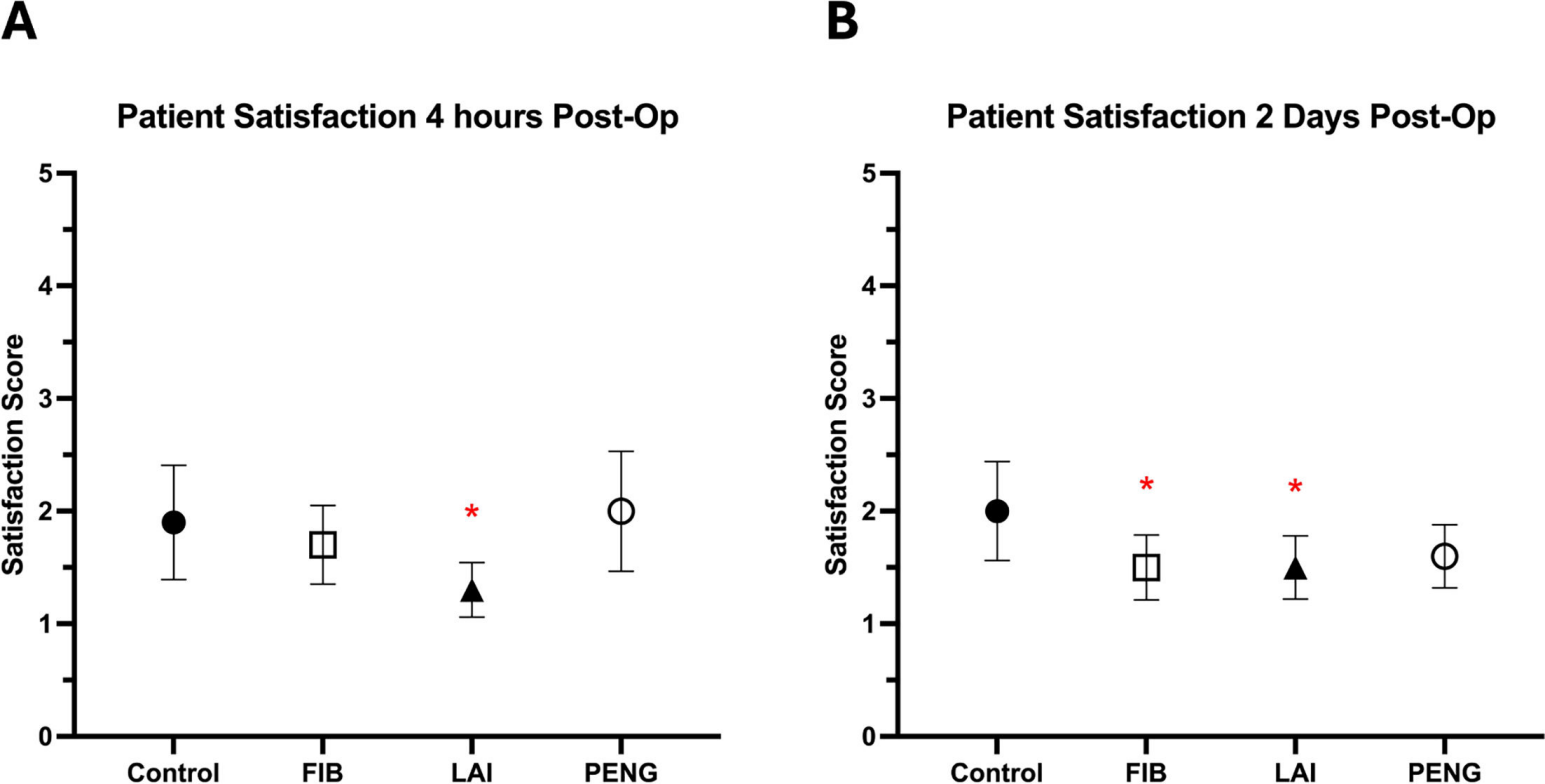
Patient satisfaction scores at: a) four hours following surgery, and b) two days following surgery. Satisfaction score was measured as 1: very satisfied, 2: somewhat satisfied, 3: neither satisfied or dissatisfied, 4: somewhat satisfied, and 5: very dissatisfied. *Statistically significant. FIB, fascia iliaca block; LAI, local anaesthetic infiltration; PENG, pericapsular nerve group block.

At four hours (Figure 4a, Table III and Table IV), satisfaction was higher in the LAI group (1.3 (SD 0.6), p = 0.043) than the control group (1.9 (SD 1.2)), while FIB (1.7 (SD 0.9), p = 0.585) and PENG block (2.0 (SD 1.4), p = 0.810) showed no difference. LAI resulted in improved patient satisfaction versus PENG block (p = 0.020). There were not significant differences between other pairs of intervention groups (FIB/LAI, p = 0.052; FIB/PENG, p = 0.414).

On POD 2 (Figure 4, Table III and Table IV), the LAI group (1.5 (SD 0.9), p = 0.042) and the FIB group (1.5 (SD 0.8), p = 0.034) had higher satisfaction with pain control than the control group (2.0 (SD 1.3)), while the PENG block showed no significant benefit (1.6 (SD 0.9), p = 0.072). There were no significant differences between pairs of intervention groups (FIB/LAI, p = 0.935; FIB/PENG, p = 0.648; LAI/PENG, p = 0.713).

There was no significant difference in patient satisfaction between individual intervention groups and the control group, or between pairs of intervention groups, before and after the first physiotherapy session and on POD 7 (p > 0.05).

### Length of stay

**Fig. 5:**
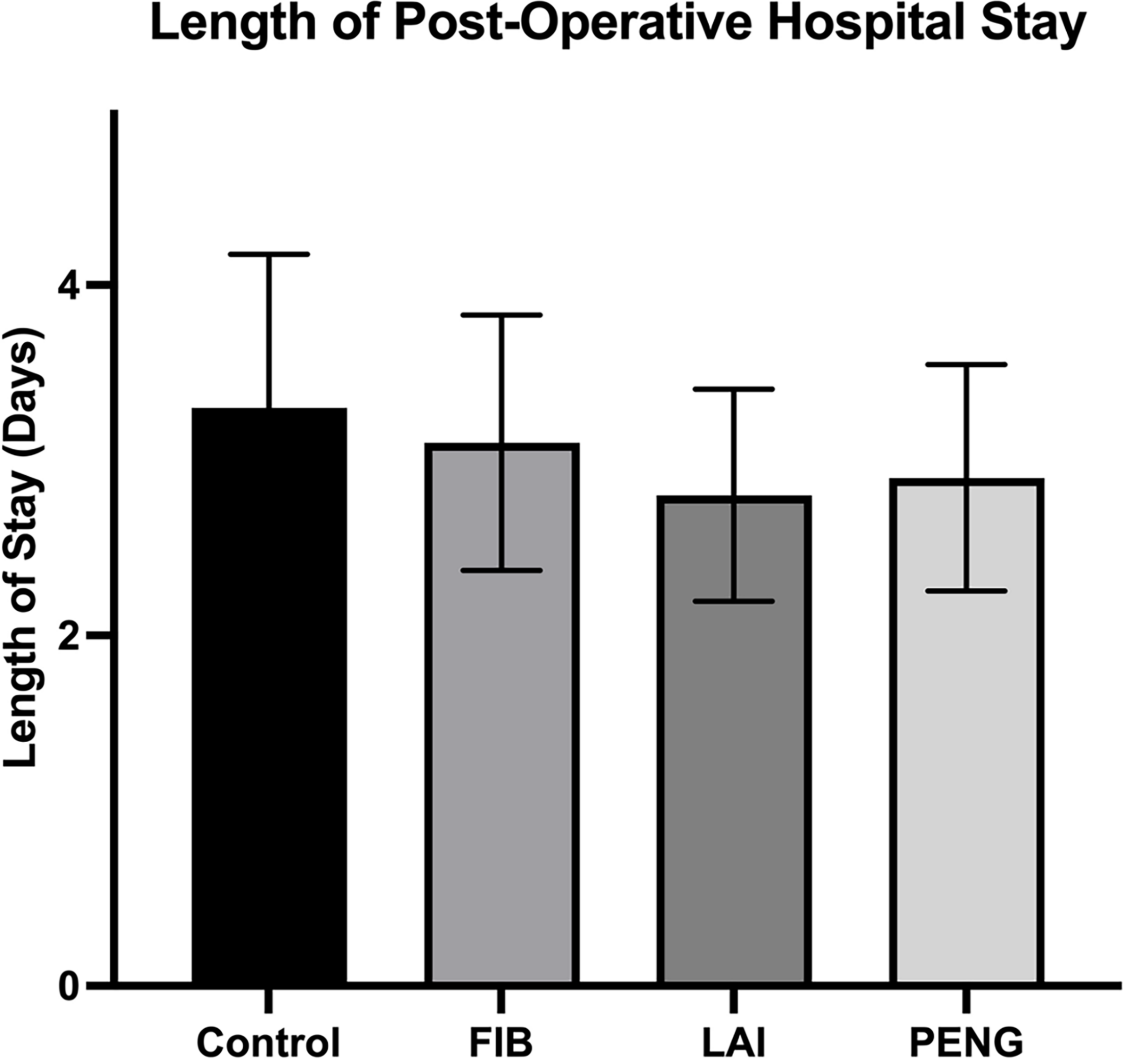
The mean length of postoperative hospital stay. FIB, fascia iliaca block; LAI, local anaesthetic infiltration; PENG, pericapsular nerve group block.

There was no difference in LOS between study groups (Figure 5). Participants in the control group had a mean LOS of 3.3 days (SD 3.3), whereas PENG block, FIB, and LAI groups had a mean LOS of 2.9 days (SD 2.3), 3.1 days (SD 2.7), and 2.8 days (SD 2.3), respectively (p = 0.433, p = 0.692, and p = 0.361, respectively, independent samples *t*-test).

### Complications

One PENG block participant had prolonged femoral nerve motor deficit, delaying their discharge from hospital. The deficit persisted beyond six weeks and was investigated with MRI and electromyography. Anaesthesia and neurology consultations led to the conclusion that this was a block-related complication. Full motor function returned by six months. No other major complications were noted (Supplementary Table ii).

## Discussion

This study compared the impact of LAI, FIB, and PENG blocks on postoperative pain following primary inpatient THA. To our knowledge, this is the first study to directly compare these analgesic adjuncts for THA. At four hours postoperatively, LAI significantly decreased VAS pain scores compared with the control group, whereas FIB and PENG block did not result in improved VAS scores. On POD 2 and POD 7, there was no difference in pain across study arms, validating available evidence that these adjuncts do not provide prolonged improvement in pain past the 24- to 48-hour postoperative period, although the available research is heterogeneous.^17,26,27^ One study found lower pain scores at 12 months following THA with the use of intraoperative LAI compared with other analgesic adjuncts, but these differences were not clinically significant.^28^ Based on our results, LAI represents an analgesic adjunct with the most effective acute pain relief following THA. Clinically, these findings contribute to the existing body of literature in this area that aid orthopaedic surgeons and anaesthesiologists in making patient-centered, evidence-informed decisions on pain control adjuncts for their patients undergoing primary elective THA.

The 1.4 cm reduction in VAS at four hours postoperatively in the LAI group did not meet the predetermined threshold for clinical relevance of 2 cm. In the design of the methodology for this trial, the 2 cm threshold was decided based on literature reporting that differences in VAS of 1.3 to 2.8 were associated with significant changes in pain, depending on the initial VAS score. However, these results were obtained from patients with isolated limb trauma with changes documented until the patient was free of pain, discharged, or a total of two hours had passed.^29^ Of note, this 2 cm threshold is greater than published VAS differences between similar pain control adjuncts. One meta-analysis comparing FIB with a control group reported mean VAS differences ranging from -0.70 to 0.35 at six hours postoperatively.^30^ Another meta-analysis comparing LAI with a control group reported standard differences in VAS ranging from -0.836 to 0.663 at 24 hours postoperatively.^31^ Other studies report similar ranges.^17,26,32-35^ One systematic review investigating 570 trials for analgesic interventions following hip or knee arthroplasty reported median minimal clinically important differences (MCIDs) for pain scores to be 15 mm at rest (IQR 10 to 20), with 46% of trials with significant primary outcomes not reaching the predetermined MCID.^36^ The authors alluded to the need for reliable evidence-based patient-rated MCIDs. Thus, the threshold for true clinical significance in postoperative VAS pain scores remains to be clearly elucidated.

While FIB and LAI both resulted in statistically significant reductions in cumulative opioid consumption compared with the control group in the first 24 and 48 hours, there was no significant difference in opioid consumption between the LAI and control groups specifically during the second 24 hours (24 to 48 hours postoperatively), suggesting that the reduction in opioid consumption by LAI occurs mostly in the first 24 hours (Table IV and Supplementary Figure a). This is consistent with the available literature that most adjuncts, but particularly LAI, decrease opioid consumption in the first day following surgery.^7,8,11,12,15,26,27^ Both FIB and LAI consisted of the same agents and dosages; thus, the shorter duration of reduced opioid consumption following LAI may relate to pharmacokinetics such as different rates of systemic absorption of the anaesthetic agents, or differences between surgeons in the method of injecting LAI in the pericapsular tissues, fascia lata, and subcutaneous tissues. Literature surrounding these specific aspects of FIB and LAI are lacking, and may be an area of interest for future studies.

In 2019, Okafor and Chen^37^ established that postoperative pain following THA is the most important factor in patient satisfaction. Our results show that at four hours postoperatively, only patients who received LAI had significantly improved satisfaction with pain control compared with the control group. Interestingly, both LAI and FIB increased patient satisfaction compared with the control group on POD 2. Similar to the reduction in opioid consumption in the first 24 hours in the LAI group, these results further suggest that LAI is most effective in the immediate postoperative period. As previously mentioned, there may be differences in pharmacokinetics or surgeon-specific application of LAI that underline these differences between LAI and FIB, however the existing literature is insufficient to validate or reject these hypotheses. Patient satisfaction with pain control is reported less frequently than pain scores or opioid consumption in this field of study. One RCT reported improved quality of recovery (QoR-15) questionnaire scores at 24 and 48 hours postoperatively with PENG block compared with spinal anaesthesia alone, while another trial reported improved patient satisfaction following FIB compared with standard intravenous analgesia.^38,39^ Finally, a review by Guay et al^40^ comparing peripheral nerve blocks, neuraxial blocks, and systemic analgesia reported low-quality evidence suggesting improved patient satisfaction with peripheral nerve blocks compared with systemic analgesia, and high-quality evidence suggesting no difference compared with neuraxial blocks. Using patient satisfaction with pain control as a reliable surrogate marker for postoperative pain, the effectiveness of LAI in the present study appears consistent with our primary outcome measure.

Our findings mirror the conclusions of previous trials. In 2021, Aliste et al^41^ reported that, for primary THA, PENG block and FIB result in comparable analgesia, opioid consumption, side effects, and LOS. However, PENG block was associated with less acute quadriceps weakness and improved hip adduction. The same group of authors compared LAI with PENG block; LAI resulted in less static and dynamic pain, with no difference in quadriceps weakness.^42^

Our study included patients admitted for inpatient postoperative care, and thus excluded primary THA patients who underwent same-day discharge, and patients having surgical indications for THA other than hip osteoarthritis. This likely biases our study population towards more medical comorbidities and/or functional deficits. Nevertheless, randomization of participants led to evenly distributed patient demographic details and clinically relevant parameters.

The difference in VAS score at four hours postoperatively between the LAI and control groups was 1.4 cm. The trial was designed to be powered to detect a 1.5 cm difference between pairs of study arms assuming a sample size of 240 patients (60 per arm) and a SD of 3 cm. Given the sample size of 222 patients in the final analysis, the study was underpowered to detect a difference of 1.5 cm.

Finally, neurological exams documented by resident physicians and nursing staff were inconsistently recorded, and therefore not presented for analysis. These findings did not delay mobilization or discharge, except in one PENG patient with motor neurological deficits persisting beyond 48 hours.

In conclusion, for inpatient primary THA, patients who receive LAI as part of a multimodal analgesic regimen experience less acute pain at four hours postoperatively (not reaching the predetermined threshold for clinical significance), reduced need for opioid consumption in the acute postoperative period, and improved satisfaction with pain control at four hours postoperatively and POD 2 compared with spinal anaesthesia alone. FIB also reduced opioid consumption up to 48 hours. PENG block has limited utility in primary THA, as evidenced by similar level of pain, cumulative opioid consumption at 48 hours, and patient satisfaction compared with spinal anaesthesia alone. The results of our study suggest that intraoperative LAI provides more benefits for pain control in primary THA for the treatment of osteoarthritis than does FIB or PENG block, when compared with spinal anaesthesia alone.

**Take home message**

- In this four-arm randomized controlled trial, local anaesthetic infltration led to significantly reduced pain, reduced opioid consumption, and improved satisfaction with pain control in the acute postoperative setting for inpatient primary total hip arthroplasty when compared with spinal anaesthesia alone.

- Fascia iliaca block conferred modest improvements in opioid consumption and satisfaction with pain control, while pericapsular nerve group block showed similar outcomes with spinal anaesthesia alone.

## Data Availability

The datasets generated and analyzed in the current study are not publicly available due to data protection regulations. Access to data is limited to the researchers who have obtained permission for data processing. Further inquiries can be made to the corresponding author.
